# Who is resilient in Africa’s Green Revolution? Sustainable intensification and Climate Smart Agriculture in Rwanda

**DOI:** 10.1016/j.landusepol.2020.104558

**Published:** 2020-09

**Authors:** Nathan Clay, Karl S. Zimmerer

**Affiliations:** a School of Geography and the Environment, University of Oxford, 34 Broad St., Oxford, OX1 3BD, UK; b GeoSyntheSES Laboratory, Department of Geography, Pennsylvania State University, University Park, PA, USA; c Department of Agricultural Economics, Sociology, and Education, Pennsylvania State University, University Park, PA, USA; d Earth and Environmental Systems Institute (EESI), Pennsylvania State University, University Park, PA, USA; e Department of Geography, Universidad Autónoma De Madrid, Madrid, Spain

**Keywords:** Agricultural intensification, Climate change, Climate Smart Agriculture, Food sovereignty, Resilience, Vulnerability

## Abstract

Under the banner of a "New Green Revolution for Africa," agricultural intensification programs aim to make smallholder agriculture more productive as well as "climate smart". As with Green Revolutions in Asia and Mexico, agricultural innovations (hybrid seeds, agronomic engineering, market linkages,and increased use of fertilizer and pesticides) are promoted as essential catalysts of agriculture-led economic growth. Intensification programs are now frequently linked to Climate Smart Agriculture (CSA), which attempts to build resilience and reduce greenhouse gas emissions while increasing crop yields. This article considers *who* and *what* is resilient in Africa’s Green Revolution. We report on a multi-season study of smallholder food producers' experiences with Rwanda’s Crop Intensification Program (CIP) and related policies that aim to commercialize subsistence agriculture while implementing CSA. . We suggest that there are fundamental limits to the climate resilience afforded by CSA and development efforts rooted in Green Revolution thinking. Our findings illustrate that such efforts foreground technology and management adjustments in ways that have reduced smallholder resilience by inhibiting sovereignty over land use, decreasing livelihood flexibility, and constricting resource access. We put forth that rural development policies could better promote climate-resilient livelihoods through: 1) adaptive governance that enables smallholder land use decision-making; 2) support for smallholder food producers’ existing agro-ecological strategies of intensification; 3) participatory approaches to visualize and correct for inequalities in local processes of social-ecological resilence Such considerations are paramount for meeting the United Nations Sustainable Development Goals and building climate-resilient food systems.

## Highlights

• Green revolution policies affect climate resilience in sub-Saharan Africa.
• Rwanda’s Crop Intensification program decreases social resilience to climatic shocks.
• The forms of resilience afforded by green revolution policies are limited.
• Sustainable intensification policies need to empower local agroecological knowledge.

## 1 Introduction

For better or worse, Green Revolution technology packets of high-yielding seeds and chemical fertilizers transformed land use and livelihoods in 20th century Latin America and Asia (Stone, 2018). Adoption of these technologies was sparse in sub-Saharan Africa (SSA), largely due to structural adjustment and fiscal austerity measures that weakened state agricultural initiatives (Denning et al., 2009). Since the mid-2000s, a resurgence of agricultural intensification programs has swept across SSA (Moseley et al., 2015). With support from World Bank, the Bill and Melinda Gates Foundation, the Rockefeller Foundation, and others, these initiatives herald a "New Green Revolution for Africa" that promises market-led agricultural transformation: commercializing agriculture to drive longterm economic growth (World Bank, 2007).

Central to the narratives legitimizing SSA's Green Revolution is a need to build climate resilience (Patel, 2013), with intensification pitched as essential to ensuring adequate food production despite projected climatic shocks and population increases in SSA (Müller et al., 2011; Lal et al., 2015;). These efforts to merge climate change with development ambitions of intensification are starkly visible in Climate Smart Agriculture (CSA), a concept developed in 2010 and championed by a wide range of development organizations including the World Bank, the Food and Agriculture Organization (FAO), and the CGIAR (Campbell et al., 2020). CSA promises a "triple win": increasing crop productivity, supporting resilience to climate change, and reducing greenhouse gas emissions (Sova et al., 2018; FAO, 2019). This article looks at linked agricultural intensification and CSA programs in Rwanda to examine *who* and *what* is resilient in Africa's Green Revolution.

Questions of "for whom" (Cretney, 2020) resilience is intended are timely given that SSA's Green Revolution comes as climate change is already impacting food security in SSA through erratic rainfall, droughts, and floods (Roy et al., 2018). The devastating locust outbreak across East Africa in 2020 has also been linked to global climate change (Stone, 2020). Even the 1.5-degree Celsius increase in global temperature anticipated by 2030 is expected to lead to periodic food shocks (Hoegh-Guldberg et al., 2018). SSA's smallholder food producers^1^ are viewed as particularly vulnerable to climate change due to their reliance on rain-fed agriculture and limited resources to cope with losses to livelihoods (Cooper et al., 2008; Schlenker and Lobell, 2010; Niang et al., 2014).

The perceived vulnerability of smallholder agriculture to climate shocks has become central to the discourse validating the present Green Revolution land use policies in SSA (Patel, 2013) and CSA has become a fulcrum to leverage a Green Revolution paradigm by attending to nebulous goals of resilence. Over the past decade, development organizations have mobilized CSA to attract emerging sources of climate change financing to support agricultural intensification (Lipper and Zilberman, 2017). As Campell et al. (2014, p39) put it, CSA "provides the foundations for incentivizing and enabling intensification." In principle, CSA intends to unite concerns of climate change and food security through sustainable agricultural transformations broadly defined (FAO, 2010). In practice, the non-specific definition of CSA means that a wide range of agricultural practices can be claimed as climate smart (Neufeldt et al., 2019). Thus, while some CSA programs might lead to robust transformations that encompass the triple-win, a singular focus on just one of these dimensions (e.g. intensification) might pervade. Indeed, the FAO notes that CSA fits well with the Green Revolution paradigm as it dovetails with existing technologies of agriculatural intensifcation through a shared emphasis on 'resource-use efficiency' and "sparing land" for ecosystem services (FAO, 2019). In Rwanda, the most recent National Agriculture Policy (covering 2017-2030) mirrors this by attempting to merge resilience and sustainable intensification in one of the four pillars for transforming agriculture (Minagri, 2017).

This article responds to calls for empirical studies on the possibilities and limits of agricultural intensification programs and CSA to promote resilience to the negative impacts of climate change as they intersect with other social-economic shocks (Rockstrom et al., 2017; Taylor, 2018). To explore *what* and *who* is resilient (Brown, 2014) in Africa’s Green Revolution, we report on empirically-generated insights from a multi-year study of rain-fed smallholder agricultural systems in Rwanda amidst the country's Strategic Plans for the Transformation of Agriculture, which is carried out chiefly through the Crop Intensification Program (CIP). Implemented countrywide since 2010, the CIP is intended to catalyze a rapid shift in land use strategies from subsistence-oriented systems premised on diverse crops to monoculture production of crops selected for their export value (MINAGRI, 2011). As with earlier Green Revolutions, this initiative is funded by international donors and coordinated through a system of checks that ensure accountability upward to the central government (Ingelaere, 2014). The CIP is guided by quintessential Green Revolution technologies (regional crop specialization, hybrid seeds, synthetic fertilizer, and land and water engineering (Pritchard, 2013)..

In many ways, Rwanda’s CIP and other ostensibly new Green Revolution land use polices in SSA can be viewed as a continuation of more than fifty years of similar efforts to intensify agriculture in Latin America and Asia through technological and policy prescriptions (Patel, 2013). Green Revolution innovations continue to emphasize agricultural technology as a way to surmount crop ‘yield gaps’ and make agriculture an engine of economic growth (World Bank, 2007; Lobell et al., 2009). Such efforts continue to rely on generalizable development outcomes that can often be far removed from the social-ecological worlds that smallholder farmers inhabit (Schurman, 2018). Yet, the greater role afforded to the private sector, which regulates smallholder producers' land use decisions through distribution of agricultural inputs and knowledge, sets SSA's new Green Revolution apart (Patel, 2013;). As a state-led project of social transformation that relies upon private-sector actors and market exchange to incentivize the adoption of high-yielding land use strategies (Cioffo et al., 2016), Rwanda's CIP is a paragon of SSA's new Green Revolution.

Sited in southwest Rwanda, a region marked by an increasing risk of drought and flood (Government of Rwanda (GOR), 2006), our analysis draws on quantitative and qualitative material from 430 household surveys, crop production data from 3017 agricultural plots across three growing seasons, and 96 in-depth interviews at household and institutional levels. The period of field research (2014–2016) encompassed two seasons of severe drought as well as several periods of torrential rains and flooding. Findings illustrate that attempts to transform agriculture in Rwanda have led to decreased food sovereignty and resilience for many, even though development programs aim to build resilience through CSA. Approximately three quarters of food-producing households—unable to bear the augmented risk of intensifying production systems by planting fewer crop varieties and relying on more purchased inputs—see their food security diminish with each successive climatic shock. With this analysis, we follow calls to consider how certain individuals and systems can become resilient and others not in contexts of social-ecological transformation (Carr, 2019; Nightingale, 2017; Turner, 2014). We advance thinking on the confines of current framings of development to confront global climate change (Nightingale et al., 2019) by drawing attention to how Green Revolution thinking sharply delimits who and what is resilient. We argue that Rwanda’s CIP and related CSA programs enforce a specific set of expert knowledge as legitimate. Further, their focus on household-level compliance disregards the need for both individual and community sovereignty and how resilience is a cross-scalar, relational process.

## 2 Analytical approach and background

### 2.1 Resilience and transformation in smallholder food production

Development policies and programs increasingly promote resilience as stability in ways that may impede desired social and economic transformation (Agrawal and Lemos, 2015; Carr, 2019). Assessing resilience--the ability to cope with and adapt to stressors--in contexts of social-environmental change necessitates attention to political economic structures, agency, and whose knowledge is considered legitimate in defining resilience (Adger and Brown, 2009; Nightingale, 2017) as well as how climate change is a lived experience that permeates livelihoods (Cote and Nightingale, 2012; Clay and King, 2019). Empirical work on social dimensions of climate change has long illuminated how vulnerability to multiple social and environmental stressors is differentially experienced among heterogeneous rural land managers (Eakin, 2005; Turner, 2016; Watts, 1983). Smallholder food producers are often ‘doubly exposed’ (cf. O’Brien and Leichenko, 2000), experiencing climatic shocks alongside other stressors such as uncertain land rights, lack of access to labor (Goldman and Riosmena, 2013; Nyantakyi-Frimpong and Bezner-Kerr, 2015), restrictive trade policies (O’Brien et al., 2004; Eakin, 2005), and processes of social reproduction and marginalization linked to race, ethnicity, and gender (Van Aelst and Holvoet, 2016; Shinn, 2016). Entrenched social inequalities are often at the root of vulnerabilities to intersecting climatic and non-climatic stressors, making grounded empirical research vital to understanding how resilience emerges in places at the interface of political economy, smallholder agency, and the biophysical environment (Ribot, 2014; Turner, 2016). To ensure that resilience is also equitable and generative of needed transformations, it is essential to consider the socio-ecological processes that shape who gets to be resilient to what (Matin et al., 2018; Carr, 2019).

Our study advances this work by interrogating the dynamics and complexities of rural livelihoods to understand how climate change is experienced within broader processes of social and environmental change (Clay, 2018; Ensor et al., 2019; Singh et al., 2017). In so doing, we also engage with recent calls to ask who is made resilient (Cretney, 2020) through dominant knowledge practices that shape the ways in which we understand the social dimensions of climate change (Goldman et al., 2018). Streamlining resilience thinking into development policies can reinforce simplistic assumptions about development outcomes in ways that carry forward existing social-ecological relations such that vulnerable people may continue to be marginalized (Carr, 2019). In this article, we ask how the knowledge and practices associated with the new Green Revolution in SSA shape particular forms of resilience. We also explore how plural forms of thinking about resilience and intensification can enable more equitable rural development.

Assessing lived experiences of climate resilience is also vital given that smallholder food producers in SSA are highly heterogeneous, operating in diverse social and agroecological conditions (Jayne et al. 2018). Smallholders in SSA pursue a range of livelihood activities along the spectrum of subsistence to commercial, often blending these strategies (Carr, 2008; Vanlauwe et al., 2014; Graeub et al., 2016; Zimmerer et al., 2015). Capacities to engage in various livelihoods are uneven, varying alongside resource access patterns that are rooted in structural inequalities and that are often attached to spatial enactments of power asymmetries (Ellis, 2000; King, 2010). As one example of this, a majority of marketable surplus typically concentrates in the hands of a few of the largest smallholders in a given area (Jayne et al., 2010). Given these uneven patterns of resource distribution, the effects of environmental variability on livelihoods tends to vary across local and regional levels (King et al., 2018). Crop viability predictions for SSA further indicate that the food security impacts of climate change will vary at the level of communities, by production system, and according to predominant crops grown, (Lobell et al., 2009; Jones and Thornton, 2009). These complexities create pressing need for community-level empirical assessments of how smallholders experience climatic shocks alongside development policies (Thornton et al., 2010; Burnham and Ma, 2016).

### 2.2 Climate resilience and SSA’s new Green Revolution

Many maintain that agricultural intensification is essential to economic growth and poverty reduction in SSA (Sanchez et al., 2009; Diao et al., 2010; Rashid et al., 2013). In bids to make Green Revolution policies relevent in a time of environmental change, some have argued further that achieving higher yields from existing farmland is necessary to curtail food shortages caused by climate change and to prevent the incursion of agriculture into land ceded for conservation (Tilman et al., 2002; Denning et al., 2009). Intensification as land-sparing is structured into CSA projects as an imperative for building climate change resilience across broader landscapes (Sova et al., 2018). This focus on land-sparing and increasing productivity through intensification is also upheld in the United Nations Sustainable Development Goals (SDGs), particularly goal 2 of zero hunger (United Nations, 2015; FAO, 2019). Legitimizing the new Green Revolution programs in SSA to support food security amid climate change requires representing earlier Green Revolutions in particular ways. As Patel (2013) notes, justifying the new Green Revolution requires representing agricultural intensification in Mexico and Asia as successful in reducing poverty and hunger. Yet, this claim of success has drawn recent scrutiny, with numerous authors demonstrating only modest gains to crop productivity that have entrenched structural inequalities and exacerbated poverty for the poorest producers (Cullather, 2013; Olsson, 2017; Perkins, 1997; Siegel, 2018).

Indeed, contemporary intensification programs, while often rebranded as sustainable intensification, appear to recreate many of these uneven effects on food security, poverty alleviation, nutrition, and the environment (Rasmussen et al., 2018). In SSA, increased crop yields and food availability have not necessarily translated to improved food access across heterogeneous smallholder social-environmental contexts (Bezner Kerr, 2013; Jayne et al., 2018; Ritzema et al., 2017). Compared to Green Revolutions in Asia, the private sector has taken a much stronger role in SSA, as decades of economic policies have weakened public agricultural research and dissemination activities (Patel, 2013). A heightened dependence upon volatile international markets also contrasts with relatively stable national markets for staple grains in 1970s Asia (Bezner Kerr, 2012). Despite the fact that climate change is leveraged as a rationale for Green Revolution-inspired agricultural intensification policies in SSA, little research has considered the viability of these programs for realizing climate resilience (Vanlauwe et al., 2014). In assessing links between climate resilience, rural livelihoods, and agricultural intensification, this article speaks to the recognized need to identify climate-resilient pathways for smallholders in SSA (Pretty et al., 2011; Lal et al., 2015; Thornton and Herrero, 2015).

### 2.3 Smallholder agriculture and intensification in Rwanda

Rwandan food producers have for generations optimized land use systems by intensively cultivating diverse crops across soil moisture regimes that are a function of space (different hill locations) and time (two rainy seasons and two dry seasons each year) (Ford, 1990). Over the years, Rwandan smallholders moved increasingly into labor-led intensification, employing mixed crop-livestock systems in which cultivation of annual and perennial crops (legumes, grains, tubers, and tree crops) is integrated with rearing livestock (cattle, small ruminants, and pigs) for manure and as capital assets (Olson, 1994). These diverse cropping strategies are often matched by diverse livelihoods that include a combination of commercial agriculture, running a small business, or other non-farm employment in trades or un-skilled industry (Bart, 1993; Clay et al., 1998; De Lame, 2005). Inequality has long been written into these variations in livelihoods and into the land itself, with soil fertility a marker of wealth (Olson, 1994).

The Government of Rwanda (GOR) has for years promoted input-led agricultural intensification with aims to commercialize smallholder production systems according to the priorities of the state and international development organizations (Newbury and Newbury, 2002; GOR 2004). In SSA’s most densely populated country, Malthusian narratives of land scarcity amid rising population have continuously underwritten these efforts. These narratives took on renewed prominence in the wake of Rwanda’s 1994 genocide. Backed by development partners such as the Bill and Melinda Gates Foundation, the World Bank, and USAID, the GOR has furthered this commitment to state-led agricultural intensification via a set of land use and rural development policies designed to turn Rwanda into a middle-income country through transforming land use in rural areas (Cioffo et al., 2016). These goals of rural transformation have increasingly begun to include language about resilience. For instance, the fourth iteration in the series of Strategic Plans for the Transformation of Agriculture intends “to strengthen resilience against the impacts of climate change and to transform the dominant subsistence farming sector into a competitive and market-led agriculture sector" (MINAGRI, 2017: 7). The chief mechanism for this shift is Rwanda’s Crop Intensification Program (CIP). The CIP has been operationalized through the World Bank's LWH project, in an effort to make intensification "climate smart" (World Bank, 2018).

Implemented countrywide since 2010, the CIP centers on increasing agricultural productivity through smallholder adoption of a technology packet: hybrid seed, increased use of agrochemicals, large-scale agro-engineering (draining marshland and constructing terraces), and expanded agriculture extension services (MINAGRI, 2011). Smallholders are compelled to consolidate land parcels and to plant government-approved crop varieties (notably maize, wheat, potato, and bean), the goal being to replace smaller family farms with larger, commercial operations that can create rural wealth and generate nonfarm employment opportunities (MINAGRI, 2012). In practice, the cultivation of certain subsistence crops (notably sweet potato, sorghum, and cassava) and the agricultural strategy of intercropping are strongly discouraged as they are seen by the state as not commercially viable (MINAGRI, 2011). Household compliance with CIP practices is enforced by local athorities, who are tasked with ensuring that quotas for converting to CIP agriculture are met. Through performance contracts (*imihigo*), households submit written agreements that they will cultivate selected crops several months before the growing season begins. Those failing to meet contracts or found to plant non-approved crops can be publicly disgraced and penalized (including fines, uprooting the crops, or jail time). An indirect result of the CIP is thus that subsistence croping strategies and key food security crops are functionally illegal in certain areas. The imposition of the CIP is, in this way, reminiscent of colonial-era polices as documented in Rwanda (Newbury and Newbury, 2002) and in East Africa (Carswell, 2006).

Although the CIP is guided by quintessential Green Revolution goals and technologies, two features make Rwanda's efforts of agricultural modernization emblematic of SSA's new Green Revolution. First, non-state and private-sector partners are responsible for the transfer of agricultural intensification technologies (hybrid seeds, synthetic fertilizer, and land engineering) to smallholders. In much of Rwanda, 'improved seeds' of government-selected crops and chemical fertilizer are delivered on credit by the One Acre Fund as part of a "bundle of services" that also includes agricultural training. Many of the terraces and irrigation infrastructure were built through the Land Husbandry, Water Harvesting, and Hillside Irrigation (LWH) project, a joint effort of the World Bank and the Rwandan Government. Secondly, climate change is invoked by each of these actors, with claims of making agriculture "climate smart." The LWH project, a 135 million USD operation that ran from 2010 to 2018, is described as having drawn on "expertise in climate-smart agriculture since 2010." The project intended "to address the critical agenda of hillside intensification through improved land husbandry" with the goal of "increasing productivity and livelihoods and reducing climate vulnerability" (World Bank, 2018). While it was not initially drawn up as a CSA project, by the end of its run the World Bank described LWH as having "Cimate-smart productive landscapes increase incomes and combat climate change" (World Bank, 2018). Likewise, the One Acre Fund describes its "core model" of market-based input and knowledge dissemination as "the ideal delivery system for our growing suite of climate smart products and services" (One Acre Fund, 2020). As we discuss below, this exemplifies how existing agendas of agricultural development can be rebranded as climate smart.

Nationally, the CIP has been hailed as a success for increasing crop yields and decreasing rural poverty (NISR, 2012). This in turn has earned Rwanda accolades and continued donor investment in the Green Revolution policies of the CIP (Harrison, 2015). Likewise, the LWH project claims to have "increased the productivity of hillside agriculture tenfold for target irrigated areas, fivefold for non-irrigated areas, and doubled the share of commercialized products (World Bank, 2018). However, several recent studies have documented crucial shortcomings of the CIP. These include inequitable provision of inputs that bypass the poorest households (Cioffo et al., 2016; Ansoms et al., 2018), impingement on smallholders’ decision-making autonomy (Van Damme et al., 2014), increased land sales by the rural poor (Dawson et al., 2016), decreased food security among the poorest groups of smallholders, and justice implications that arise when the households that are least able to succeed with the CIP are the most compelled to adopt it (Clay, 2017). The present article builds on these assessments by considering climate resilience amid the CIP. Empirical material on lived experiences of vulnerability amid climate variability and shocks provides valuable insight into potential future responses to comparable fluctuations brought by global climate change (Engle and Lemos, 2010; Turner, 2016). We argue below that the CIP synergizes with the LWH and market-led model of One Acre fund in that all three frame resilience as a supply-side issue of increasing land use effiency. This framing entrenches reliance upon technological and managerial changes that may afford limited potential to ensure equitable development and climate risk management.

## 3 Study sites and methods

### 3.1 Study sites

This research was conducted in four *umudugudu* (rural settlements) in Nyamagabe district. Located in southwest Rwanda, Nyamagabe (formerly Gikongoro) has historically been among Rwanda’s poorest regions. As elsewhere in Rwanda, a feudal system structured land use in the pre-colonial era—with *Tutsi* owning land and grazing cattle and *Hutu* practicing agriculture on rented land—and persisted through the colonial period. As much of the land was reserved for pasture, agriculture intensified and colonial land use policies sought to avoid land degradation through building terraces and erosion ditches (Gourou, 1953,b; Harroy, 1944). Agriculture further intensified amid rapid population growth following independence. Given sparse off-farm employment opportunities in Gokongoro, soil fertility management techniques developed locally, including tree planting, leasing additional fields, caring for others’ animals for manure, and collecting grasses for green manure. The uneven availability of these resources contributed to a widening soil fertility gap between rich and poor and between men and women (Olson, 1994).

Present-day Nyamagabe has been an early target of the GOR’s land use intensification policies and a recent government survey (GoR EICV, 2016) indicates that poverty has reduced dramatically over the past five years. Rwanda’s National Adaptation Plan of Action (GOR, 2006) categorizes Nyamagabe as a region with high occurrence of both drought and heavy rain. Smallholder agriculture is the principal livelihood for nearly all households in the region, with rain-fed production tied to two annual growing seasons (one from September to December, the other from March to May). Some households diversify livelihoods with non-farm employment (e.g. small business, trades such as masonry or house framing, and labor such as collecting sand or making charcoal) and by selling commercial crops in addition to subsistence. The CIP has been active in the study communities since 2010 and has been adopted by 90 percent of households.

### 3.2 Methods and background findings

Qualitative and quantitative methods were conducted at farm-plot, intra-household, household, and institutional levels. A household survey on resource access, livelihoods, decision-making, and climate risk management was conducted by a team of five research assistants with all available households in the four communities n = 428. A parcel-level survey was conducted with the 402 households with operational holdings n = 3017 agricultural parcels, examining biophysical aspects of fields as well as inputs, crop yields, and impacts of climate shocks. Each survey was pilot tested with more than 25 respondents. Surveys were conducted by four teams of two research assistants. Research assistants were experienced in conducting social surveys and underwent two days of training for this project. Crop yields were reported by respondents based on local methods of measuring, which were converted to kilograms. Semi-structured interviews were conducted by the first author and two research assistants with 36 male and 36 female respondents selected randomly. The average interview time was 1 h 20 min. Field notes were taken and the three interviewers met immediately after to verify accuracy. Interviews were also conducted with key informants and local and regional administrators (n = 38, purposively sampled).

Data collection spanned from June 2014 to December 2016, a period coinciding with severe rainfall aberrations and shocks across the country (Nahayo et al., 2018). Our analysis combines assessment of livelihoods and resource access with the impacts of climatic shocks on crop yields (at the farm-plot level) and with effects on food security and coping strategies (at the household level). Qualitative analysis was conducted using ATLAS.ti, coding for key issues related to climatic vulnerability, livelihood change, power dynamics, and agricultural intensification. Quantitative analysis was carried out using SPSS. A two-step cluster analysis (hierarchical and non-hierarchical) was employed to classify smallholder households into four groups according to livelihood attributes: assets (land, livestock, consumer goods), principle livelihood, and income. The composite livelihood and asset indicator variables are compared in Table 1. The results of this analysis produced four clusters of smallholders that are used in some of the statistical tests. These clusters are of course imperfect, as many households cross over into various categories at different times. Yet these groups also enable analysis of differential impacts of land use policies. From analysis of variance (ANOVA), these groups showed significantly different values across all five indicators (p < .01; two-tailed ANOVA). According to respondents, these indicators are important components of agricultural livelihoods in the study communities. We enrich the discussion of these groups with insights from analysis of qualitative interviews. The four clusters are named according to principal livelihood and are described as follows:

• *Agricultural laborer* households (primarily agricultural labor on neighbors’ farms, land average 0.12 ha, most have no livestock, little or no income)
• *Resource-poor agricultural* households (primarily cultivating own farm, most own one pig, sheep, or goat, or rent a bull, land average 0.29 ha, little income)
• *Diversified agriculture + NFE* households (many combine cultivation with nonfarm employment (NFE) work or business, average 0.67 ha land, most own one cow, modest income)
• *Commercial agriculture + business* households (most combine cultivation with NFE or business, average 1.57 ha of land, most own two or more cows, substantial income)

**Table 1:** Composite livelihood and asset indicators used to group households in cluster analysis. Bi-variate ANOVA finds significant difference between groups for all indicators.

Livelihood & assets group	N	Land (mean ha)	Farm-labor income (%)	Non-farm income (RWF)	Cows (mean)	Small livestock (mean)	Female household head
Ag laborers	117	0.12	91.9	5252	0.1	0.5	37.6%
Resource-poor ag	137	0.29	69.4	21,268	0.3	0.8	20.4%
Diversified ag + other	128	0.65	52.9	147,576	0.6	1.2	15.6%
Commercial ag + business	49	1.54	34.4	611,676	1.3	2.1	8.2%
All groups	431	0.51	71.8	120,640	0.5	1.0	22.3%
Sig.	–	0.000	0.000	0.000	0.000	0.000	0.000

## 4 Results

### 4.1 Mitigating climatic variability, uncertainty, and change

**Fig. 1:**
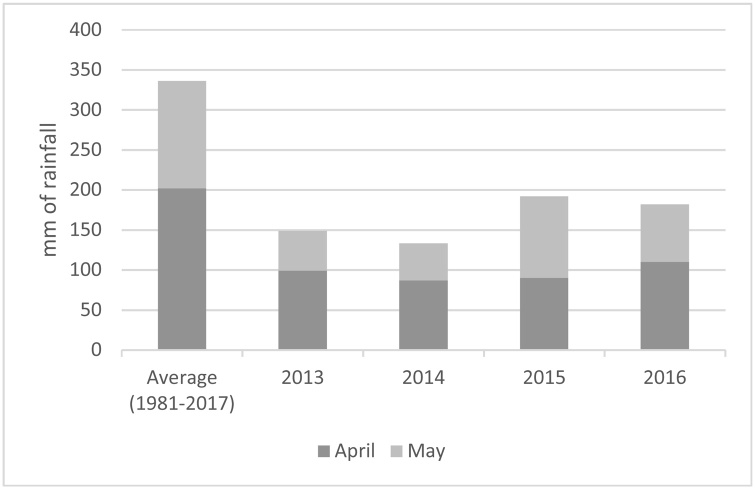
Recent rainfall anomalies in Nyamagabe, Rwanda, compared to 1981-2017 averages. Data from Meteo Rwanda.

Immediately before and during the 2014–2016 study period, numerous extreme weather events occurred in Rwanda. In the March-May growing season of 2013, rainfall was far below average (FEWS, 2013) and in 2014, this rainy season ended two months earlier than expected. This effectively cut the growing period in half and substantially reduced yields for most crops. This dry spell was felt throughout East Africa and across Rwanda, particularly in the south and east of the country, with impacts on maize being especially severe due to the crop’s long maturation period (FEWS, 2014). During the subsequent growing season (September to December 2014), late rain onset followed by unusually heavy rains ten weeks into the season (a key moment of crop development) negatively impacted crop yields, particularly for beans, which are easily damaged by torrential rain. Similarly, in both the 2015 and 2016 March-May growing seasons there was below-average rainfall and an early end to rains, leading to devastating losses across the country (FEWS, 2016), particularly for maize (Nahayo et al., 2018). Together, these events added up to what Rwanda’s Ministry of Agriculture called “the longest drought in six decades” (New Times, 2016). In Nyamagabe, where important April and May rains combine to an average of 332 mm (1981–2017), these months saw less than half of the average between 2013 and 2016 (Fig. 1). Reflecting this, a majority of study participants reported that climate has changed since they have lived in the area. Of 428 surveyed, 65 percent remarked that there is now more uncertainty as to when rain will begin and when it will end, while 70 percent noted increased uncertainty in the total amount of rain each season.

When asked about the optimal food production strategies to counter uncertain rainfall and extreme climate events, respondents pointed to the importance of diverse agricultural systems. As Table 2 shows, 72 percent of respondents surveyed said that it is better to plant many different food crops in a season than to plant one or a few crops. Over 90 percent said that they prefer to plant in various fields across a variety of locations (including upper hillside, mid-hillside, lower hillside, valley, and marsh). This indicates that while both land use diversification strategies are important, planting in numerous fields is considered an especially valuable mechanism to mitigate the risk of losses due to uncertain climate dynamics. Interview participants also pointed to the importance of preparing land ahead of time so that crops can be planted immediately following the season’s first rains, thereby minimizing risk of failed harvest if the growing season ends earlier than expected. Interviewees consistently emphasized the importance of choosing a crop based on the biophysical attributes of their fields, explaining that soil fertility and moisture regimes vary substantially over short distances depending on slope, position on the hillside, and history of cropping and fertilizing. So too did they discuss the importance of planting key food security crops—sweet potato and cassava—in times of climatic distress.

**Table 2:** Perceptions of climate changes and land use strategies to mitigate risks of unpredictable weather (n = 428).

Rainfall amount uncertain	Climate has changed since here	Many crops better	Many fields better
70 %	65 %	72 %	91 %

To quantify views on climate-resilient crops, survey respondents were asked to list the three best and three worst crops to grow under drought and heavy rain scenarios. As shown in Table 3, beans, peas, maize, potato, and wheat are widely viewed as the riskiest. These five crops account for 91 percent of those listed as worst for drought and 88 percent as worst for rain. Sweet potato, cassava, and banana are the least risky (these crops amount to 76 percent of those selected as the best crops for drought and 55 percent of the best for heavy rain). This gives a clear picture of which crops are seen to fare the best and the worst in the face of climatic shocks. This divide starkly points to the CIP crops as riskier and underscores the importance of crop selection as a smallholder mechanism of risk mitigation that is performed alongside decisions based on soil fertility.

**Table 3:** Respondent perceptions of best and worst crops for drought and flood. Households listed up to three best and three worst crops (totals differ because some respondents listed fewer than three).

	Best Crops				Worst Crops			
Crop	Drought		Rain		Drought		Rain	
	Number	Percent	Number	Percent	Number	Percent	Number	Percent
None	17	1.7%	18	1.7%	2	0%	1	0%
Beans	20	2.0%	34	3.2%	395	37%	380	36%
Peas	5	0.5%	14	1.3%	172	16 %	188	18 %
Maize	64	6.4%	287	27.3 %	161	15 %	34	3%
Wheat	38	3.8%	40	3.8%	129	12 %	110	10 %
Sweet Potato	311	31.3%	236	22.4%	2	0%	14	1%
Cassava	245	24.7%	177	16.8%	8	1%	21	2%
Sorghum	3	0.3%	17	1.6 %	8	1%	2	0%
Banana & Taro	191	19.2%	145	13.8%	6	1%	9	1%
Potato	41	4.1 %	18	1.7%	121	11 %	229	21%
Tea	13	1.3%	7	0.7%	6	1%	3	0%
Woodlot	34	3.4%	42	4.0%	0	0%	0	0%
Horticulture	11	1.1%	18	1.7%	65	6%	76	7%
Total	993	100.0%	1053	100.0%	1076	100 %	1067	100 %

### 4.2 Agricultural intensification and extreme climate events

**Fig. 2:**
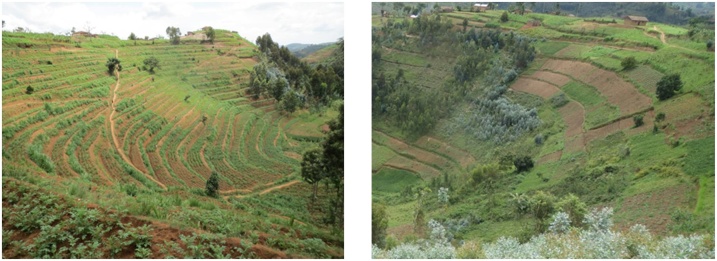
Examples from study site: strictly consolidated land on terraces (image on left) compared to less strictly consolidated land (image on right). Photos by author, from October 2014.

Various aspects of Rwanda's land use policies, although folded into CSA programs by LWH, the One Acre Fund, and Rwanda's Government, have undermined these strategies for the mitigation of climate risk in food production. As other studies have pointed out, the intercropping of multiple crop species and varieties is seen by many as a valuable risk mitigation mechanism (Isaacs et al., 2016; Zimmerer, 2010). Yet this practice is directly prohibited under the CIP. Although still practiced clandestinely, study participants emphasized that intercropping has all but disappeared in the area. When the research team occasionally noticed intercropping, respondents would often laugh, explaining that contemporary intercropping is scarcely what it used to be. The intercropping ban is enforced by local authorities who are known to tread into fields uprooting the prohibited crops. Secondly, planting in many different fields is discouraged by the land-use consolidation component of Rwanda’s CIP (as discussed in Section 2.4), wherein all fields in a given area of the hill must be planted in the same crop variety. The goal of standardizing in this way is to streamline input use to increase crop yields and create economies of scale. In practice, land use consolidation has led to fewer separate fields, particularly in areas where bench terraces have been constructed and where marshlands have been drained, as it is in these places where land use consolidation is most strictly enforced (Fig. 2). This land use engineering is offered by the LWH as CSA, yet clashes with locally-embedded strategies of mitigating climate shocks, namely planting many different fields and across micro-variations in soil-water regimes.

Consolidation is most strictly enforced on terraces given the substantial financial investment in terrace construction. Third, under the CIP, cropping decisions are made by government authorities—again especially for terraced areas. This weakens food-producers’ capacities to utilize the variable soil and moisture regimes within and between fields. Fourth, the government-approved crops require far greater amounts of purchased inputs (including seeds, fertilizer, and pesticides), which are seen by study participants to further exacerbate the risk of financial loss in the event of climatic shocks. Respondents frequently spoke about the decreased resilience to climatic shocks that has resulted from the prohibition of food-producing polycultures. For example: *‘Yes, they have changed completely… in past years we mixed crops so even if a disaster comes there was little problem, but now we have to plant only one crop…if a disaster comes it is all destroyed.’* By locking producers into crop rotations based on regional and national targets, the overall climate resilience of communities is thus reduced. This occurred throughout Rwanda, particularly with maize, which requires rainfall in the second and third month of growth (Nahayo et al., 2018). Maize is the most important CIP crop in terms of area planted, even though it is not traditionally consumed in Rwanda. This calls into question the claims of climate smart agriculture made by the One Acre Fund, which supplies bundles of services that include fertilizer and seeds for high-yielding varieties of maize and beans in the study site in line with the government's mission of agricultural commercialization.

The effects of the CIP were widely felt in the study community, as 90 percent of surveyed households altered land use in accordance with the program in 2015. Study participants indicated that they had little choice in whether to abide by these policies (74 percent claimed that their involvement was not voluntary). Moreover, 75 percent of all households (including those not consolidating land) said that the government makes cropping decisions for them. This loss of autonomy presents another challenge in that crops selected by the authorities (bean, maize, wheat, peas, and potato) are seen to fare the worst in drought and heavy rain scenarios (as discussed in section 4.1). Interview and survey respondents regularly noted the negative impacts on their food security, explaining how food-production systems once hinged on flexible decision-making that enabled them to match crops to the heterogeneous agro-ecological conditions of their landholdings and to ever-changing household needs. Moreover, interview participants expressed powerlessness to adaptively respond to changing climatic conditions. When asked whether she would alter her farm management practices by planting different crops after recent climate shocks decimated her maize and bean crops, a resource-poor woman responded: *‘I will not change because I cannot change. Local leaders told us to grow these crops, even though some years drought occurs and other years there is heavy rain.’*

**Fig. 3:**
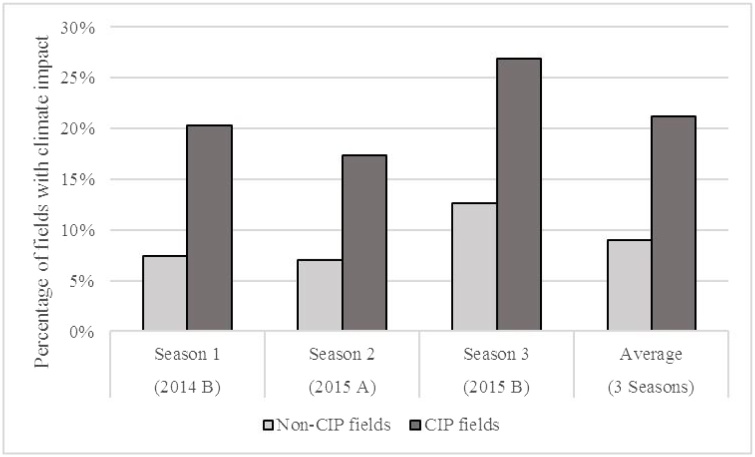
Effect of land consolidation and CIP crops on field susceptibility to yield losses from climatic events in three growing seasons (2014-2015).

These preferences for diverse farming systems and low-risk crops are consistent with crop yield data collected at farm-plot level across three growing seasons. We compared the incidence of yield-reducing climatic shocks on the range of crops planted on various field types (fields planted because of the crop intensification program and those planted without influence of the program). While on CIP fields one of the government-selected crops was grown, a key feature of non-CIP fields was that traditional (prohibited) crops were more likely to be grown (often sweet potato, local rice, or cassava). Concerning these two field types and climate shocks, statistical analyses reveal that in fields planted under the government’s crop intensification mandate (CIP fields) there is significantly higher occurrence of yield reduction than non-CIP fields. As shown in Table 4 and Fig. 3, yield-reducing climatic challenges (drought, heavy rain, or late start/early end of growing season) had a significant negative impact on the yield of CIP fields during each of the three seasons. These yield losses resulted in livelihood setbacks that negatively impacted food and nutritional security. Averaged across three seasons, 21.2 percent of CIP fields experienced climate-related yield reduction compared to 9.0 percent of non-CIP fields.

**Table 4:** Comparison of CIP and Non-CIP fields in terms of climatic shocks that reduced crop yields across three seasons.

Field Type	(2014 B)	(2015 A)	(2015 B)	(3 Seasons)
	Season 1	Season 2	Season 3	Average
Non-CIP fields	7.4 %	7.0 %	12.6 %	9.0 %
CIP fields	20.3 %	17.3 %	26.9 %	21.2 %
All fields (mean)	10.8 %	10.6 %	16.5 %	12.6 %

This differential was especially pronounced in Season 2014B (March to June 2014), when CIP fields exhibit a threefold higher incidence of yield-reduction from climatic shocks in comparison to non-CIP fields. This is likely due to the occurrence of drought during that season, which had a large negative impact in fields planted with maize or wheat under CIP specifications, as these are crops that households less commonly choose to plant on their own. By contrast, Seasons 2 and 3 of the study were marked by late onset of rain and then periods of heavy rain, which respondents pointed out had a particularly negative impact on bean and peas, crops that households do grow traditionally, outside of government cropping mandates. The fact that Season 3 has the highest occurrence of climatic shocks likely owes to a combination of late start to rain (which had a major impact on wheat) followed by heavy rains (which impacted bean and peas).

Table 5 further clarifies these dissimilarities and complements survey findings concerning perceptions of the differential climatic risk of the eight most cultivated food crops. Fields of cassava, sorghum, and sweet potato all exhibit relatively low occurrence of significant yield losses from drought and heavy rain across the three growing seasons (5, 6, and 9 percent, respectively). By comparison, fields of wheat, maize, bean, peas, and potato have a much higher occurrence of yield losses due to extreme weather events (18, 19, 27, 30, and 34 percent, respectively). The variability of climate impacts across crops also emerges in terms of crop value per hectare. Table 5 quantifies yield losses due to climatic shocks by comparing crop values per hectare of fields with climatic shocks with fields where no climatic shocks occurred. Overall, these findings suggest that in compelling households to cultivate selected crops amid uncertain rainfall, Rwanda’s agricultural intensification policies have introduced greater risks into smallholder systems, making them more susceptible to the negative effects of extreme weather events.

**Table 5:** Impact of climate shocks on yields of principal crops grown in study site.

Crop	Drought or Dry Spell				Heavy Rain			
	Crop value (RWF)--No shock	Crop value (RWF)--Shock	Percent value change	Proportion of fields impacted	Crop value (RWF)--No shock	Crop value (RWF)--Shock	Percent value change	Proportion of fields impacted
Bean	152,779	101,060	−34%	11 %	152,779	101,175	−34%	16 %
Peas	111,217	86,497	−22%	14 %	111,217	56,082	−50%	16 %
Maize	184,336	94,988	−48%	15 %	184,336	92,619	−50%	4%
Wheat	195,936	138,156	−29%	12 %	195,936	202,775	3%	6%
Potato	221,580	122,891	−45%	16 %	221,580	169,734	−23%	18 %
Sweet Potato	506,272	288,192	−43%	8%	506,272	221,157	−56%	1%
Cassava	270,647	301,604	11 %	3%	270,647	288,458	7%	2%
Sorghum	112,869	26,250	−77%	3%	112,869	28,530	−75%	3%
Banana	555,014	983,457	76%	9%	555,014	177,127	−68%	1%

### 4.3 Decreased social resilience to extreme weather events

This study finds that elevated climatic risks are unevenly experienced across households, with implications for social resilience. Households showed differential vulnerability to climatic risks, which relates to deeply-rooted social inequalities, which shape resource entitlements and livelihood and land use options. The 2014 drought is a prime example of this. Across the 428 households surveyed, harvests were cut nearly in half by rains ending early (47.6 percent mean yield loss). Yet this varies across the four study *umudugudu*. In the community where the CIP is least enforced, median household losses from drought were only 40 percent, compared to losses of more than 50 percent in the other three communities. The overall impacts of the CIP on household coping capacity is further evident in that 30.1 percent of all households reported lower ability to mitigate the 2014 drought compared to previous droughts. Moreover, this change in drought coping capacity varies significantly by household livelihood group. Among households with the lowest resources, who generally have little land and rely on laboring in others’ fields for income, 39.6 percent said their ability to cope with the drought had decreased, compared with only 25 percent of the wealthiest households (Table 6). On the other hand, 14.6 percent of wealthier commercial-level producers reported increased ability to cope, compared to only 2.8 percent and 1.6 percent of the two lowest-resource groups. Households reporting decreased coping ability emphasized the difficulty of finding work given the low demand for unskilled labor. This contrasts with those observing that their coping ability had increased, who tended to have steady incomes from consistent wage work in nonfarm employment (e.g. masonry) or business.

**Table 6:** Change in household coping capacity according to livelihood and asset group.

Livelihood & asset group	Drought coping ability			
	Decrease.	Increase	Net change	
Ag Laborers	39.6 %	2.8 %	−36.8	2.8
Resource-poor ag	26.8 %	1.6 %	−25.2	1.6
Diversified ag + other	27.3 %	4.1 %	−23.3	4.1
Commercial ag + business	25.0 %	14.6 %	−10.4	
Total	30.1 %	4.2 %	−25.9	4.2
Sig.	0.002	0.002		

A second aspect of households’ differential vulnerability to climatic shocks lies in the intersections of climate and access to crucial agricultural resources (notably inputs of fertilizer, manure, seed, and labor). Qualitative analysis of interviews reveals dynamics between climate risks and willingness to invest in fields. Respondents frequently noted that while maize, bean, and wheat can produce substantial yields, that is only feasible when climatic conditions are ideal and adequate manure and chemical fertilizer are applied. Households with limited access to resources experience these intersecting challenges to a much greater degree than do those with enough resources to buffer losses from unpredictable climate. Quantitative data further illustrate the complexities of how this differential vulnerability to intersecting losses from low fertility and climate shocks is experienced. For example, during the drought of 2014 the disparity between advantaged and disadvantaged households experiencing intersecting challenges of soil fertility and climate in non-CIP fields was only 9.4 percent, while for CIP fields the gap jumped to 27.1 percent. In other words, while drought can affect all fields, these exogenous climatic shocks can interact with endogenous challenges of differential resource access—which are rooted in structural inequalities. This illustrates how Rwanda’s CIP, in combination with uneven access to productive resources, has shaped household vulnerability to climatic shocks. The result is a situation where the disparity among households in terms of vulnerability to climatic shocks has become aggravated by the CIP.

The exacerbating of differential resilience to climatic shocks is further evident in the emerging disparities among socioeconomic groups in terms of changes in agricultural production in recent years. Table 7 summarizes survey results on the direction of yield changes, displaying percentages of respondents who report that household crop yields had increased or decreased as compared to two years prior and five years prior to the study. These time periods are instructive for different reasons. The ‘five years’ period captures changes associated with Rwanda’s CIP, which was widely adopted in the community four years prior to the study. The ‘two years’ period captures changes specifically related to climate shocks that occurred in 2014 and 2015 in addition to effects of the longer-term policy changes. As Table 7 shows, compared to two years before the study, net yield change was negative for nearly 47 percent of all households, hovering around 50 percent for the lowest three livelihood/asset groups. These findings suggest strong negative impacts of climate shocks for all but the wealthiest commercial agriculture households. Yield changes compared to five years prior to the study reveal an even starker discrepancy: large yield decreases for a majority of households among the most marginalized groups compared with only modest net declines among the more advantaged groups. This divide reflects the effects of top-down agricultural intensification measures. Together, these findings further illuminate the intersecting effects of climate shocks, resource access differentials, and agricultural intensification policies in resilience to climatic shocks.

**Table 7:** Effect of livelihood/asset group on change in agricultural yield. Results from chi square.

Livelihood and asset group	Change in agricultural yield (two years)			Change in agricultural yield (five years)		
	Decrease	Increase	Net change	Decrease	Increase	Net change
Ag Laborers	53.5 %	3.0 %	−50.5%	64.0 %	14.0 %	−50.0%
Resource-poor ag	55.0 %	4.6 %	−50.4%	65.0 %	18.0 %	−47.0%
Diversified ag + other	61.6 %	10.4 %	−51.2%	58.0 %	28.0 %	−30.0%
Commercial ag + business	45.8 %	27.1 %	−18.7%	49.0 %	43.0 %	−6.0%
All groups	55.6 %	8.6 %	−47.0%	61.0 %	23.0 %	−38.0%
Sig.	0.000	0.000		0.002	0.002	

## 5 Discussion and policy implications

The above results shed light on who and what is resilient in SSA’s ongoing Green Revolution and in CSA projects that are now frequently linked to programs of intensification. Efforts in Rwanda to standardize the production of key export crops at scale through constructing terraces and irrigation infrastructure, consolidating land, and encouraging the adoption of hybrid seeds and fertilizer have intersected with structural inequalities to introduce substantial climatic risks despite claims that such programs are "climate smart." The form of resilience afforded in these Green Revolution projects hedges on balancing production out over time and space. There is an assumption that even if some fields or communities are unproductive in some years, overall productivity gains in other regions and in other years will offset these losses. Yet, only the wealthiest of study participants, who have substantial land and alternative cash flows, proved resilient to the series of droughts, unpredictable starts to growing seasons, and bouts of torrential rain that occurred between 2013 and 2016. The most marginalized groups, including those with little land and female-headed households, experienced decreased ability to cope with climatic shocks. This form of resilience has fundamental limits in terms of social equity. Rwanda's agricultural development program ensures that national-level food security and economic growth are prioritized over local or intra-household livelihoods.

What appears most resilient in SSA's new (and allegedly climate smart) Green Revolution is the vision of agricultural development itself: market-led rural transformation through increasing crop yields at the national level. In Rwanda, this Green Revolution model is upheld by the development initiatives of the World Bank and the One Acre Fund, which align closely with the government's ambitions for rural transformation through technoligical and managerial changes. As the Project Completion Report for LWH describes it, the key "factors driving the success of the project" were that the "Government and the Bank were dedicated to a sector-wide rural development and other donors were willing to support LWH's program without variance in project implementation features" (Oblitas, 2019; IEG Review Team, 2019). This illustrates the powerful inertia of a Green Revolution vision to generate compliance with one-size-fits-all agricutlural development rather than a responsive system of checks and balances among the multiple organizations. By practicing agricultural intensification through the language of CSA, these organizations have established themselves as brokers of climate change resilience in ways that give further credence to technological expertise, bolstering the power of external actors to make decisions that impinge on the land use and livelihoods of rural communities. This model bodes poorly for hoped-for rural poverty reduction in a time of global warming when climate shocks such as occurred during this study will likely become increasingly pronounced (Niang et al., 2014). Resilience, according to the new Green Revolution and CSA programs, amounts to outsmarting climate change through reliance on antiquated bundles of technology and expert knowledge.

As demonstrated in this article, negative vulnerability outcomes can arise when smallholders’ risk mitigation strategies are devalued while they are simultaneously forced to adopt riskier commercial crops in lieu of traditional food security crops during a period of increasing climatic unpredictability. If intensification policies such as Rwanda’s CIP continue to be implemented and enforced in SSA in the context of increasingly uncertain climates in the region, the lives and livelihoods of tens of millions of smallholders will be threatened, potentially resulting in a setback or even a reversal in the outcomes promised from agricultural intensification policies. For intensification policies to create opportunities for more resilient smallholder agriculture, these findings suggest the importance of fostering flexibility and autonomy at community levels. Specifically, agricultural governance measures must be decentralized and must be adaptive enough to account for the spatially and temporally heterogeneous capacities of smallholders to mitigate climate shocks. Specific attention must be given to the social dimensions of vulnerability to climatic shocks, and the diverse and potentially uneven ways that shocks are mitigated via livelihood and land use strategies. This will likely be especially crucial in mountainous areas, where substantial micro-variations in the effects of climate change on food production are predicted across a topographically and socioeconomically heterogeneous region (Thornton et al., 2010). More generally, the findings presented in this article underscore the need to approach agricultural intensification and climate resilience as complex political ecological issues.

Rwanda’s status as a perceived development success (Harrison, 2015) gives the country an opportunity to create and showcase what an effective agricultural policy might look like under climate change. Although accountability currently leans heavily towards the central government, Rwanda’s robust governance structures extend to local levels (Ingeleare, 2014). If reworked to democratically disperse power and enable more local autonomy and flexibility, this strong network could foster adaptive governance (c.f. Shinn, 2016), with reflexive, context-based decision-making enabling greater smallholder resilience in the face of global climate change. However, such a policy environment will require a wholesale reformulation in the way that agricultural intensification is conceptualized such that enduring social inequalities in rural Rwanda--legacies of decades of coercive state-society relationships (Newbury and Newbury, 2002)--are actively overturned rather than reconstituted. Rwanda's current Strategic Plan for the Transformation of Agriculture and National Agricultural Policy seem to offer little in the way of such transfomation as these plans both frame climate resilience as dependent on intensification and commercialization of agriculture. A more climate-wise (Taylor, 2018) approach to intensification might recognize the intersections among climate change, deeply-rooted social inequality, and rural transformation.

How might agricultural intensification policies and CSA be reformulated in ways that build more inclusive rural resilience? Towards this aim, this study responds to calls for empirical insight to guide reformulation of growth-oriented development policies vis-à-vis multiple components of risk (Agrawal and Lemos, 2015). In illustrating how intensification policies can shape smallholder vulnerabilities in unexpected ways, this research points to the importance of reformulating policies with explicit consideration of risk management mechanisms and concern for how governance changes might contribute to increasing agricultural productivity without jeopardizing the capacity of farm households to mitigate climate risks. To ensure food security amid variable and uncertain climate in SSA, we argue that it is essential to enable the households least able to successfully adopt large-scale intensification to opt out of intensification programs such as the CIP. We suggest further that it is important to consider the potential viability of multiple pathways of intensification, including locally preferred intensification strategies that may be labor led and rely on local ecological knowledge and available resources. In Rwanda, intercropping, cultivating across multiple moisture and temperature gradients, and planting diverse cultivars are three such strategies that enable flexible and resilient production systems. Rwanda's land use policies, particularly on terraces constructed through the LWH program and other donor-funded initiatives, heavily restrict these practices and result in outcomes that appear contrary to both climate resilience and productivity-enhancing goals of CSA. Nevertheless, the LWH program is upheld by the World Bank as "another proof of concept of climate smart agriculture to achieve significant results even in the most challenging environments" (World Bank, 2018).

To ensure smallholders’ climate risk mitigation abilities, we suggest that agricultural intensification policies during a time of rapid climate change would be wise to incorporate *agroecological* intensification strategies. which support biodiversity and are already widely practiced by smallholders in SSA (Tomich et al., 2011; Zimmerer, 2010). Within SSA, agroecological mechanisms such as intercropping maize and legumes are shown to improve biodiversity, ecosystem function, and crop yield over monoculture in Malawi (Snapp et al., 2010) and Rwanda (Isaacs et al., 2016). Such mechanisms have the potential to enhance resilience in the face of climatic variability and extreme events (Lin, 2011; Waha et al., 2013). Indeed, agrobiodiversity at farm, field, and crop genetic levels has been demonstrated to be an important component of the resilience of smallholder agriculture (Zimmerer, 2010; Tomich et al., 2011; Waha et al., 2013; Zimmerer et al., 2015).

Agro-ecological intensification necessitates valuing local environmental knowledge—which Green Revolution policies have consistently undermined (Patel, 2013). Studies throughout SSA have demonstrated the importance of local knowledge that smallholders employ to mitigate climatic risks (Ingram et al., 2002; Roncoli et al., 2002; Gamble et al., 2010; Orlove et al., 2010). For example, in their study of seed aid in Ethiopia, McGuire and Sperling (2008) found that this supply-side development program ignored local climate risk management strategies and marginalized local knowledge of drought-tolerant seed varieties. This work connects with the burgeoning discussion on agrobiodiverse farming systems, where local environmental knowledge and practices are essential to maintaining diverse crop cultivars (Snapp et al., 2010; Schipanski et al., 2016; Meldrum et al., 2017; Zimmerer et al., 2015). Our findings resonate with this work. We echo calls for these strategies to be incorporated into land use policies in hopes of enabling more just and resilient livelihoods in this time of global climate change (Rockstrom et al., 2017). This is particularly important for programs at the scale and extent of Rwanda’s CIP.

## 6 Conclusion

This study examined the forms of resilience enabled in agricultural intensification programs that aim to promote anew Green Revolution for SSA that is also "climate smart". The Rwandan government's vision of input-led agricultural intensification as a pathway to economic growth was upheld through the World Bank's LWH agricultural engineering project and the One Acre Fund's input distribution model. While these organizations claim that their operations in Rwanda adhere to CSA principles, in practice this has granted further authority to expert technical knowledge and international organizations as arbiters of resilience. Meanwhile, top-down agricultural intensification in Rwanda has resulted in the unanticipated consequence of decreased resilience to climate shocks for a large segment of smallholder food producers in this study. Not only has this form of intensification disabled endogenous farming and risk management practices, it has compelled smallholder food producers to cultivate crops that may be poorly suited for their land and their levels of resource access, particularly their ability to purchase inputs. As a result, many respondents experienced decreased agricultural productivity and decreased food security. This reflects the inability of Green Revolution land-use policies to effectively buffer risks. It also exemplifies how top-down intensification programs can overide the aims of resilience espoused by CSA with a singular focus on maximizing crop yield. This resonates with admonitions that the vagueness of CSA principles opens the concept to co-optation by powerful entities and existing agendas (Campbell et al., 2020; Taylor, 2018). .

Green Revolution thinking, in the case of Rwanda, has permeated CSA platforms such that rural development operations emphasize economic growth at the expense of climate risk management in the land use systems of smallholder food producers. The has particularly negative impacts on the poorer segments within this group, who have felt a decline in their capacity to cope with climate shocks as they have been forced to pursue strategies that further drain their already scarce resources. These results bolster calls for agricultural intensification policies that are attuned to the social and ecological heterogeneities that characterize land use systems in SSA (Thornton et al., 2009; Clay, 2017). Moreover, this research adds empirical clarity to calls for making risk management a central policy goal of agricultural development (Agrawal and Lemos, 2015) in general, and sustainable intensification specifically (Loos et al., 2014). This is particularly necessary for approaches premised on intensification, which can bring further risks to already vulnerable smallholder populations.

For Rwanda, this means that rather than mandating agricultural intensification uniformly across space and time, it is essential to consider options for a nuanced approach that recognizes that there are various groups and areas of land use for whom the risks associated with full compliance are simply too high. For SSA, it is essential to consider how resilience to climate change is experienced in places and how vulnerability emerges differentially across complex and heterogeneous social-ecological landscapes. Methodologically, this study has demonstrated the value of integrating data from farm-plot, household, and institutional levels to consider the complex effects of land use and policy changes on smallholder resilience to climate change. Further research on these issues can benefit from a place-based approach to the intersections of climate resilience, inequality, and land use policies. To those hoping to implement a "climate smart" Green Revolution, this study urges caution. As the Rwanda case demonstrates, CSA can be mobilized towards one-size-fits-all development solutions. And the World Bank is now upholding the LWH project as a "proof-of-concept" for its CSA activities (World Bank, 2018). Care must be taken to ensure that other agricultural intensification and CSA programs do not reinforce top-down directives for productivity increases at the expense of other goals of sustainable development. Above we discussed how agricultural intensification could support more equitable resilience through 1) enabling adaptive governance that empowers smallholder producers and 2) validating community understandings of climate change and resilience and taking seriously smallholder food producers' existing strategies of agroecological intensification and risk-magangement. It is further essential to employ participatory approaches when designing and implementing agricultural intensification and CSA. While participatory approaches are far from a panacea, they can be a first step in visualizing how power differentials and intersecting stressors may shape who gets to be resilient. Attention to these issues in agricultural intensification policies and programs is vital for working towards the United Nations SDGs and for equitably building climate-resilient food systems.

## Funding sources

This fieldwork on which this article is based was funded by the Fulbright Program of the Institute of International Education, a United States Agency for International Development (USAID) Borlaug Fellowship for Global Food Security. Analysis and writing were supported by the Wellcome Trust, Our Planet Our Health (Livestock, Environment and People – LEAP), award number 205212/Z/16/Z.

## Declaration of Competing Interest

The authors have no conflicts of interest to disclose.

## Footnotes

1 Those in the smallest quintile of landholding, generally less than two hectares of land (Lowder et al., 2016), but in Rwanda this would be less than ½ hectare.
